# Alexithymia and asthma: a systematic review

**DOI:** 10.3389/fpsyg.2023.1221648

**Published:** 2023-08-07

**Authors:** Orlando Silvestro, Luisa Ricciardi, Antonino Catalano, Carmelo Mario Vicario, Francesco Tomaiuolo, Giovanni Pioggia, Giovanni Squadrito, Peter Schwarz, Sebastiano Gangemi, Gabriella Martino

**Affiliations:** ^1^Department of Health Sciences, University Magna Graecia of Catanzaro, Catanzaro, Italy; ^2^Department of Clinical and Experimental Medicine, University Hospital of Messina, Messina, Italy; ^3^Department of Cognitive Sciences, Psychology, Education and Cultural Studies, University of Messina, Messina, Italy; ^4^Institute for Biomedical Research and Innovation (IRIB), National Research Council of Italy (CNR), Messina, Italy; ^5^Department of Endocrinology, Research Centre for Ageing and Osteoporosis, Rigshospitalet-Glostrup Hospital, Copenhagen, Denmark

**Keywords:** alexithymia, asthma, psychosomatic disorders, psychological distress, chronic disease, clinical psychology

## Abstract

Growing evidence from scientific research elucidates the important role of alexithymia in chronic immune diseases. This Review aims to explore the presence of alexithymia in patients affected by asthma and clarify its associations with other involved psychological and physical factors. In January 2023, according to PRISMA guidelines, a systematic search using PubMed and Scopus was conducted. Twenty-six studies were eligible based on inclusion criteria. Alexithymia was significantly present in asthma patients, with most studies reporting a higher prevalence (from 9 to 62.8%) than in control groups (approximately 10%). The coexistence of asthma and alexithymia was associated with a worse quality of life, psychiatric comorbidity, poor symptom control, and difficulty in recognizing exacerbations of the disease. These results suggest that alexithymia can negatively impact the management of asthma. For this reason, we recommend an accuracy assessment in clinical settings and the implementation of psychological interventions to promote the emotional and physical wellbeing of asthmatic patients.

## 1. Introduction

Over time, researchers have shown increasing interest in the psychological factors that influence the onset, course, and outcomes of chronic illness (Cohen et al., 2015; Martino et al., 2019; Eikeseth et al., 2020; Bąk-Sosnowska et al., 2022). Psychological characteristics may influence the patient’s ability to manage chronic disease and contribute to determining variable health outcomes (Van Lieshout and MacQueen, 2008; Merlo, 2019; Conversano et al., 2020; Di Giuseppe and Conversano, 2022). On the other hand, the management of medical conditions represents a complex challenge and could increase the risk of developing psychopathology (Baiardini et al., 2015; Di Giuseppe et al., 2020; Isvoranu et al., 2021; Lin et al., 2022).

Asthma is a chronic respiratory disease characterized by persistent airway inflammation and represents one of the major public health issues worldwide (World Health Organization, 2019; Mortimer et al., 2022). It is characterized by heterogeneous symptoms, including shortness of breath, airway secretion, wheezing, chest pain, and coughing attacks, which are variable over time and in terms of intensity (Braido, 2013; Louis et al., 2023). For this reason, the Global Initiative for Asthma (GINA) guidelines classifies asthma, based on the frequency and severity of symptomatic manifestations, as intermittent and persistent, and the latter is subdivided into mild, moderate, or severe. In addition, a second classification criteria refers to the level of symptom control and allows differentiation between well-controlled, partially controlled, or uncontrolled asthma (Padem and Saltoun, 2019; Global Initiative for Asthma, 2022). According to epidemiological data provided by the “Global Burden of Disease Study,” it is estimated that 262 million people (cases: 3,416 per 100,000 people) presented with asthma in 2019 (GBD 2019 Diseases and Injuries Collaborators, 2020). Mortimer et al. (2022) analyzed the prevalence of asthma in adults in 17 countries and highlighted a rate of 4.4%; the authors provided results obtained from a study called “Global Asthma Network,” including a huge sample of adults showing a mean prevalence of 2.6% of asthma symptoms. According to the study, asthma represents a heavy burden for patients and relatives, impacting on quality of life and adaptation, as well as development. Hence, asthma as a complex medical condition causes significant social and economic burdens (Bahadori et al., 2009; Nunes et al., 2017; López-Tiro et al., 2022).

The control of asthma symptoms is complex and influenced by the presence and interaction of several factors (Braido, 2013), such as phenotypes (Hsiao et al., 2019), comorbidities (Boulet and Boulay, 2011), type of treatment (Papi et al., 2020), and patient characteristics, such as sociodemographic factors (Uchmanowicz et al., 2016), compliance (Baddar et al., 2014), adherence (Boulet et al., 2012), and behaviors (Horne et al., 2007). Moreover, different psychological aspects contribute to the process of asthma adaptation and management (Baiardini et al., 2015; Stanescu et al., 2019). Subjective perception of illness (Kaptein et al., 2010), coping strategies (Barton et al., 2003), presence of anxiety, and/or depression (Di Marco et al., 2011; Ye et al., 2021) strongly impact daily asthma management and consequently the main areas of life functioning, determining a poor perceived quality of life (Van Lieshout and MacQueen, 2008; Stanescu et al., 2019).

Alexithymia is a psychological factor reported in patients suffering from chronic respiratory diseases, such as asthma and chronic obstructive pulmonary disease (COPD), and is related to negative effects on quality of life and the exacerbation of symptoms (Braido, 2013). According to different studies, alexithymia has been described as impacting, complicating, and often directly affecting the psychological condition of subjects (Baiardini et al., 2015; Myles and Merlo, 2021). Selinheimo et al. (2022) and Zhang et al. (2023) highlighted a bidirectional influence between alexithymia and chronic respiratory diseases, and thus, reported that alexithymia increases the risk of developing chronic airway inflammation and vice versa. In the first study, subjects were followed for alexithymia for 11 years using binary logistic regressions to investigate the role of psychological factors in the onset and maintenance of physical conditions. In the second, a consistent decrease in quality of life in chronic respiratory diseases due to alexithymia was highlighted.

According to the literature, alexithymia is a multifactorial construct, defined as the difficulty in recognizing, processing, and expressing emotions and feelings, resulting in the inability to differentiate between physiological reactions to stimuli and affective dynamics (Sifneos, 2000; López-Muñoz and Pérez-Fernández, 2020). The term alexithymia was introduced by Nemiah and Sifneos (1970) to describe a lack of knowledge referring to affective dynamics in patients with psychosomatic disorders resistant to traditional treatments. Since then, this concept has undergone wide diffusion in the scientific literature, moving from its psychoanalytic origin to research fields on mechanisms of stress adaptation (Panayiotou, 2018; Myles and Merlo, 2021). In this regard, alexithymia has been described as a combination of different factors (Bagby et al., 1994; Hogeveen and Grafman, 2021), such as difficulty in identifying and describing affective states, difficulty in distinguishing kinesthetic sensations related to emotional arousal, reduced ability to imagine and fantasize, and an external event-oriented thinking style. These features have been frequently recognized in psychosomatic disorders, demonstrating that alexithymia is implicated in the alteration of the body–mind communication axis (Taylor et al., 1997; Kano and Fukudo, 2013). Moreover, in several chronic conditions, a high prevalence of alexithymia was observed among patients with negative health outcomes, also increasing the risk of developing anxiety and depression (Tselebis et al., 2010; Nekouei et al., 2014; Martino et al., 2020a,b; Zhang et al., 2023).

According to Tesio et al. (2019), alexithymia is clearly linked to precise medical domains, as in the case of dermatological diseases (Panasiti et al., 2020; Namdar and Kurtoglu, 2021; Holmes et al., 2022), cardiovascular conditions (Casagrande et al., 2019; Aluja et al., 2020; Kirchner et al., 2022), gastrointestinal disorders (Carrozzino and Porcelli, 2018; Kano et al., 2018), and chronic respiratory conditions such as asthma.

Currently, a range of studies have linked alexithymia to asthma; however, there has been no systematic synthesis of the available research examining this association. Probably because of this reason, there are several unanswered questions regarding this relationship, including whether alexithymia is frequently present in individuals with asthma and whether the characteristics of alexithymia can specifically interfere with asthma management.

The aim of this study was to perform a systematic literature review on alexithymia and asthma, offering new insights for application into clinical practice. Particularly, the present review was based on two main objectives: to provide a summary of the available evidence on the presence of alexithymia among individuals with asthma and the factors associated with alexithymia in asthmatic patients. A deeper understanding of this complexity may promote psychological adjustments, improve disease consciousness and management, and decrease the risks of adverse health outcomes.

## 2. Methods

This systematic review has been performed in accordance with the Preferred Reporting Items for Systematic Review and Meta-analyses (PRISMA) guidelines (Liberati et al., 2009; Moher et al., 2009). The search methodology and related steps are presented as follows.

### 2.1. Search strategy

In January 2023, PubMed and Scopus databases were explored to select relevant publications. The terms searched were: “asthma” AND “alexithymia” [title, abstract, and keywords] (see Table 1 for the complete PubMed strategy). The same strategy was adopted for both databases without restrictions related to language and time. The reference lists of suitable studies from both databases were examined with the aim of identifying additional relevant articles to be added to previously selected items.

**Table 1:** List of terms entered in the PubMed and Scopus search.

Number	Term
1	ASTHM* [all fields]
2	ALEXITHYMIA [all fields]
3	TORONTO ALEXITHYMIA SCALE [all fields]
4	TAS-20 [all fields]
5	2 OR 3 OR 4
6	1 AND 5

### 2.2. Inclusion and exclusion criteria

**Figure 1:**
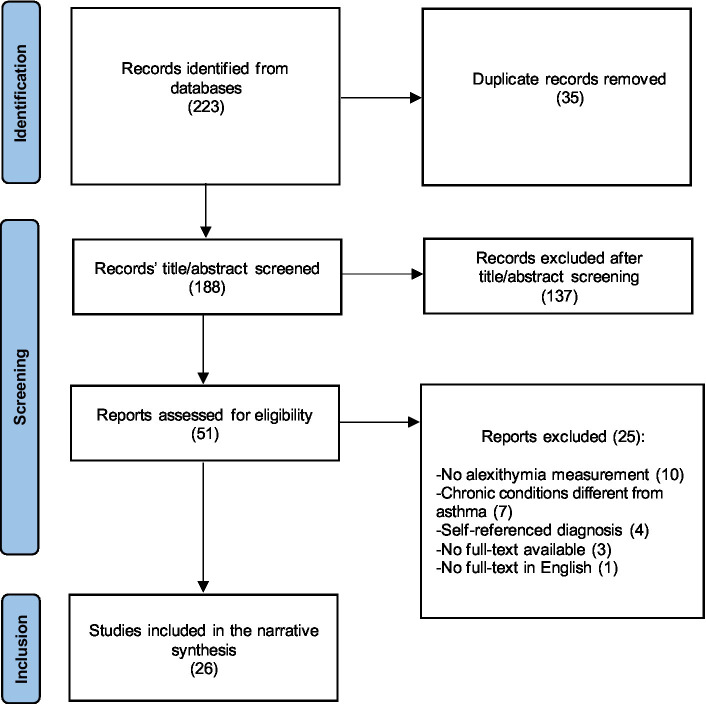
Preferred Reporting Items for Systematic Review and Meta-analyses (PRISMA) flow diagram.

Once the PubMed and Scopus search was performed, the eligibility of the articles was based on the following criteria: (1) full-text available in English and published in peer-review journals to reduce the risk of including biased studies; (2) confirmed asthma diagnosis; and (3) use of standardized psychodiagnostic instruments measuring alexithymia to detect the variables of interest. In line with the purpose of this Review, the following publications were excluded: (1) conference abstracts, qualitative research, literature reviews, and case reports, selecting only comprehensive studies with adequate statistical methodology to link asthma to alexithymia; (2) studies in which the diagnosis was self-reported, ensuring accurate selection of the asthma population; and (3) articles in which the sample included respiratory chronic conditions other than asthma or mixed diagnosis, not being able to consider the direct relationship between asthma and alexithymia (see Figure 1, Prisma flowchart).

### 2.3. Screening and data extraction

After extracting results from databases, a thorough screening procedure was carried out to eliminate duplicates. In the first phase, all titles and abstracts were screened to identify potential pertinent articles. During the second phase, full-text manuscripts were examined and selected according to eligibility criteria. Data extracted from this research included author names, publication date, sample size and characteristics, main results, and tool used for alexithymia measurement.

### 2.4. Assessment of the methodological quality of the included studies

A complete evaluation of the studies was performed using the NIH Study Quality Assessment Tool. Each study was evaluated according to structure and study design by two independent reviewers, and two more reviewers were consulted when necessary. Specifically, the Quality Assessment Tool for Observational Cohort and Cross-sectional Studies, the Quality Assessment Tool for Before-After (Pre-Post) Studies with No Control Group, and the Quality Assessment of Controlled Intervention Studies were used. As shown in Table 2, this revision included 22 cross-sectional studies and six observational cohort studies evaluated with the first tool, one study assessing the effectiveness of a pharmacological intervention without a control group, and one randomized controlled trial. Finally, all the included studies were rated as “good” or “fair,” and outcomes were presented as Supplementary Table S1–S3.

**Table 2:** Characteristics of included studies.

Authors	Year	Study design	Country	Sample	Measure	Main findings
Amore et al.	2013	Observational cohort	Italy	117 patients with BA	TAS-20	A subgroup of patients with high levels of alexithymia and low pulmonary function (FEV_1_) who reported lower symptom control scores and more maladaptive coping strategies than the group with low alexithymia and higher lung function. The two groups reported no differences in anxiety and depression variables. Furthermore, alexithymia was positively correlated with the severity of anxiety and depression.
Baiardini et al.	2011	Cross-sectional	Italy	115 patients with asthma and comorbid rhinitis	TAS-20	19% of the sample reported high levels of alexithymia scores. These subjects presented lower levels of asthma control and worse quality of life than non-alexithymic subjects. Furthermore, patients with alexithymia referred to more negative effects of asthma and rhinitis and reported higher levels of depression and stress, with negative consequences on disease management.
Barbosa et al.	2011	Cross-sectional	Portugal	53 patients with systemic lupus erythematosus 41 patients with asthma	TAS-20	A high percentage of alexithymia in both groups. No significant differences were found between the groups, either in total TAS-20 scores or in single dimensions of the instrument (DIF, DDF, and EOT).
Brown et al.	1981	Cross-sectional	United States	136 asthmatic patients with alexithymia 134 asthmatic patients without alexithymia	MMPI Alexithymia Scale	Asthmatic individuals with alexithymia minimized reporting their somatic and affective symptoms, showing lower scores than asthmatic subjects with low alexithymia scores.
Chugg et al.	2009	Cross-sectional	Australia	25 patients with asthma	TAS -20	Alexithymia scores were elevated in 12% of the sample associated with poor asthma management, adherence, and worse quality of life; alexithymia did not influence the level of satisfaction and physician-patient communication.
Dafauce et al.	2021	Cross-sectional	Spain	63 patients with severe asthma	TAS-20	A large portion of the sample reported high TAS-20 scores (42.9%). Older age, depression, and/or anxiety (HADS) were more present in asthmatic patients with alexithymia. Furthermore, subjects with hyperventilation syndrome showed higher TAS-20 and HADS scores. Quality of life was worse in asthmatic patients with alexithymia, depression, anxiety, and hyperventilation syndrome.
Dirks et al.	1981	Observational Cohort	United States	579 patients with asthma	MMPI alexithymia scale	A strong association between alexithymia and rehospitalization as patients with asthma and alexithymia showed a higher rate of rehospitalization.
Feiguine et al.	1982	Cross-sectional	United States	474 patients with asthma	MMPI alexithymia scale	Alexithymia was associated with the age of the participants. Results showed higher levels of alexithymia in middle (43%) and late (60%) adulthood than in adolescents and early adulthood.
Feldman et al.	2002	Cross-sectional	United States	74 patients with asthma	TAS-26	Subscale DIF was associated with emotional and physical symptoms, but not with pulmonary function. Subscale DDF was associated with decreased pulmonary function. EOT was not associated with other measures.
Ghorbani et al.	2017	Cross-sectional	Iran	300 patients with asthma 100 control group	TAS-20	Patients with asthma reported higher scores in all dimensions of TAS-20, as well as non-adaptive emotion regulation strategies, than the control group. DIF, DDF, and EOT were associated with physical symptoms indirectly, through catastrophizing.
Innamorati et al.	2015	Cross-sectional	Italy	153 BA patients	TAS-20	22% presented severe airway obstruction and 51% reported higher levels of alexithymia. These subjects reported more depressive symptoms and higher self-reflectiveness.
Khosravani et al.	2016	Cross-sectional	Iran	300 patients with asthma 100 control group	TAS-20	Higher alexithymia scores and maladaptive emotion regulation strategies than the control group. Patients with alexithymia presented higher asthma severity and physical symptoms than other subjects affected by asthma. Three TAS-20 factors and maladaptive emotion regulation strategies predicted physical symptoms.
Khosravani et al.	2020	Cross-sectional	Iran	300 patients with asthma from mild to severe	TAS-20	Higher levels of alexithymia in the sample (47%). DIF factor was associated with high levels of negative affectivity and low empathy. Alexithymia influenced physical symptoms.
Kleiger and Dirks	1980	Observational cohort	United States	202 patients with asthma	MMPI alexithymia scale	Both alexithymia and panic-fear personality scale scores were associated with a higher number of rehospitalizations. These patients showed more difficulty in adherence to treatment in the opinion of clinicians. Alexithymia and panic-fear personality traits had no association with pulmonary function measured in the long-term.
Kleiger and Jones	1980	Cross-Sectional	United States	76 patients with asthma 16 COPD 8 tuberculosis	BIQ SSPS	Assessment with the BIQ showed high levels of alexithymia in all groups. Asthmatic subjects with high levels of alexithymia had lower psychasthenia and higher scores on the lies (L)-scale assessed with MMPI.
Liotta et al.	2021	Before-after (pre-post) studies with no control group	Italy	18 patients with severe allergic asthma	TAS-20	6 of 18 patients presented high levels of alexithymia. All subjects reported an improvement in asthma control and symptom exacerbation rates during omalizumab treatment.
Martínez-Rivera et al.	2011	Cross-sectional	Spain	264 patients with asthma 111 control group	TAS-20	In the asthma group, dysfunctional breathing was higher than in the control group. These patients presented higher alexithymia and anxiety, poorer control of asthma symptoms, and worse quality of life than asthmatic patients without dysfunctional breathing.
Moes-Wójtowicz et al.	2012	Cross-sectional	Poland	54 patients with asthma	TAS-26	Alexithymia was present in 21.6% of cases. This variable was not associated with stress, strong emotions, and asthma symptom control.
Nielsen et al.	1997	Cross-sectional	Canada	76 patients with asthma	TAS-26	Different results by gender. High TAS scores, especially EOT subscale, in men was associated with low dream recall, regardless of age and neuroticism. In women participants dream and nightmare recall was correlated only with neuroticism.
Plaza et al.	2006	Observational cohort	Spain	50 NFA patients 25 non-NFA patients 25 non-asthmatic control	TAS-26	Higher alexithymia scores in the NFA group (24%) than in the non-NFA (12%) and non-asthmatic control groups (12%). The mean (SD) TAS score was 63.6 (14.9%). Poor ventilatory responses and breathlessness perception were not associated with alexithymia. The presence of alexithymia was associated with a greater number of hospitalizations in the past.
Serrano et al.	2006	Observational cohort	Spain	179 Near Fatal Asthma (NFA) patients 40 non-NFA patients, as a control group	TAS-26	Higher alexithymia scores in NFA subjects than in non-NFA ones. Higher age percentage in patients with NFA and elevated alexithymia, together with more psychopathological issues, lower education, and a higher proportion of persistency and symptom exacerbation.
Smyth et al.	2002	Controlled intervention study	United States	39 patients with asthma 32 patients with rheumatoid arthritis	TAS-20	High levels of alexithymia and non-expressive characteristics were not associated with an inability to write about traumatic experiences for both groups.
Vanegas et al.	2020	Cross-sectional	Latin America (multicentric)	265 patients with asthma	TAS-20	High levels of alexithymia were reported in asthmatic patients (30.2%). Lower levels of education were correlated with the presence of alexithymia. Subjects with high alexithymia scores had more uncontrolled and severe asthma.
Vázquez et al.	2010a	Cross-sectional	Spain	44 NFA patients 44 non-NFA patients	TAS-20	Higher levels of anxiety and alexithymia in patients with NFA than in the non-NFA group. These patients reported higher scores on DDF subscales and higher psychopathological onset risk than patients from the control group, even years after the asthma attack.
Vazquez et al.	2010b	Cross-sectional	Spain	76 patients with asthma	TAS-20	DIF was correlated with physical components of quality of life, DDF was associated with the number of hospitalizations. EOT was not related to any of the dependent measures.
Vliet et al.	2002	Observational cohort and cross-sectional	United States	30 multiple chemical sensitives 19 patients with asthma 31 healthy controls	TAS-26	Levels of alexithymia were similar among groups. Subjects with asthma and Multiple Chemical Sensitivities (MCS) showed no differences from the control group.

BA, Bronchial Asthma; FEV_1_, Forced Expiratory Volume in the 1st second; TAS, Toronto Alexithymia Scale; DIF, Difficulty Identify Feelings; DDF, Difficulty Describing Feelings; EOT, External-Oriented Thinking; U.S., United States; MMPI, Minnesota Multiphasic Inventory; HADS, Hospital Anxiety and Depression Scale; COPD, Chronic Obstructive Pulmonary Disease; BIQ, Beth Israel Hospital Psychosomatic Questionnaire; SSPS, Schalling-Sifneos Personality Scale; NFA, Near Fatal Asthma.

## 3. Results

The PRISMA flowchart (Figure 1) illustrates the search strategy used for this systematic review. Through the databases search, 223 potentially eligible studies were identified and 35 were removed as duplicates. The remaining 188 records were reviewed with regard to title and abstract, and 137 were removed because of low accordance with the objectives of this Review or due to exclusion criteria. The process of recognizing valuable items through analysis of the reference lists produced no further results. Thus, 51 studies were comprehensively reviewed and 25 were excluded according to eligibility criteria. Finally, a total of 26 records were included in the current Review and analyzed with regard to three main domains (Table 2): the study characteristics and the tools used to assess alexithymia; the presence of alexithymia traits in subjects affected by asthma; and alexithymia correlation with psychological factors and physical components interfering with asthma.

### 3.1. Characteristics of the included studies

All 26 studies presented participants suffering from asthma, leading to a total of 4,282 subjects. Fifteen studies included adults (Feldman et al., 2002; Smyth et al., 2002; Vliet et al., 2002; Chugg et al., 2009; Vázquez et al., 2010a; Vazquez et al., 2010b; Baiardini et al., 2011; Barbosa et al., 2011; Martínez-Rivera et al., 2011; Innamorati et al., 2015; Khosravani et al., 2016, 2020; Vanegas et al., 2020; Dafauce et al., 2021; Liotta et al., 2021) and eight also included adolescents (Kleiger and Dirks, 1980; Kleiger and Jones, 1980; Dirks et al., 1981; Feiguine et al., 1982; Nielsen et al., 1997; Serrano et al., 2006; Amore et al., 2013; Ghorbani et al., 2017). Finally, three studies (Brown et al., 1981; Plaza et al., 2006; Moes-Wójtowicz et al., 2012) did not provide clear data on the age range of participants.

Asthma severity and intensity differed within the selected study. Specifically, eight studies (Brown et al., 1981; Feiguine et al., 1982; Nielsen et al., 1997; Feldman et al., 2002; Smyth et al., 2002; Vliet et al., 2002; Barbosa et al., 2011; Moes-Wójtowicz et al., 2012) selected subjects with asthma of varying degrees and did not report data on disease severity. Four studies (Khosravani et al., 2016; Ghorbani et al., 2017; Khosravani et al., 2020; Vanegas et al., 2020) analyzed patients affected by chronic asthma, with severity ranging from mild to severe. Additionally, Martínez-Rivera et al. (2011) included participants with intermittent asthma. Dirks et al. (1981), Kleiger and Dirks (1980), and Kleiger and Jones (1980) involved hospitalized patients receiving treatment. Chugg et al. (2009) and Vazquez et al. (2010b) analyzed subjects presenting moderate-to-severe persistent asthma. Two studies (Amore et al., 2013; Innamorati et al., 2015), conducted in Italy, included a sample of subjects diagnosed with bronchial asthma (BA). Three studies (Plaza et al., 2006; Serrano et al; 2006; Vázquez et al., 2010a) selected subjects affected by near-fatal asthma (NFA). Finally, Baiardini et al. (2011) analyzed 115 patients affected by asthma and comorbid rhinitis, Dafauce et al. (2021) conducted a study on 63 subjects with severe asthma, and Liotta et al. (2021) reported a small sample consisting of 18 patients with severe allergic asthma.

All studies used a standardized instrument to assess alexithymia. TAS-20 was used in more than half of the studies (Smyth et al., 2002; Chugg et al., 2009; Vázquez et al., 2010a; Vazquez et al., 2010b; Baiardini et al., 2011; Barbosa et al., 2011; Martínez-Rivera et al., 2011; Amore et al., 2013; Innamorati et al., 2015; Khosravani et al., 2016, 2020; Ghorbani et al., 2017; Vanegas et al., 2020; Dafauce et al., 2021; Liotta et al., 2021), followed by the previous 26-item version of the instrument (Nielsen et al., 1997; Feldman et al., 2002; Vliet et al., 2002; Plaza et al., 2006; Serrano et al., 2006; Moes-Wójtowicz et al., 2012). Four studies (Kleiger and Dirks, 1980; Brown et al., 1981; Dirks et al., 1981; Feiguine et al., 1982) measured alexithymia as a personality trait through the MMPI subscale. Only one single case (Kleiger and Jones, 1980) used a hetero-assessment interview (BIQ) and a self-report instrument (SPSS) to compare the homogeneity of the collected data.

Three studies included a group of asthma patients and a healthy control group (Martínez-Rivera et al., 2011; Khosravani et al., 2016; Ghorbani et al., 2017). Two studies compared patients with asthma with other chronic diseases, such as systemic lupus erythematosus (Barbosa et al., 2011) and rheumatoid arthritis (Smyth et al., 2002). Vliet et al. (2002) examined differences among subjects with multiple chemical sensitivities, asthma, and healthy controls. Regarding studies analyzing subjects with asthma and episodes of NFA, Serrano et al. (2006) and Vázquez et al. (2010a) included asthmatic subjects who presented no near-fatal exacerbations of disease as control groups, while Plaza et al. (2006) also included a third group of healthy subjects.

#### 3.1.1. Instruments used for alexithymia assessment

Several instruments allow clinicians to measure alexithymia, but the TAS-20 (Bagby et al., 1994; Parker et al., 2003; Bagby et al., 2020) is regarded as the “gold standard.” This instrument analyzes three factors: difficulty identifying feelings (DIF); difficulty describing feelings (DDF); and externally oriented thinking (EOT). As known, DIF and DDF indicate an inability to become aware of affective states, denoting subjective experience of feelings (Goerlich, 2018), whereas EOT derives from the psychoanalytic literature, defined as “*pensée opératoire*” (Marty and de M'uzan, 1978), and indicates a way of thinking directed toward external objects, devoid of imagination and fantasy (Pirlot and Corcos, 2012; Schmid-Gloor, 2019; Mambou Nouemssi et al., 2021), as in the case of regression in the service of the ego (Kris, 1952).

Cross-validation studies demonstrated high consistency and validity of the TAS-20 scale, improving its scientific consideration and clinical-psychodiagnostic impact (Bach et al., 1996; Bressi et al., 1996; Franz et al., 2001; Torres et al., 2019; Bagby et al., 2020). It is derived from the previous version, the TAS-26, assessing alexithymia by dividing the construct into four factors (Taylor et al., 1985), presenting a scale for assessing the reduction in the ability to daydream. As the concept of reduced daydreaming revealed poor psychometric properties and little theoretical coherency with the alexithymia construct, it was substituted with the TAS-20 (Wise et al., 2000; Bagby et al., 2020).

As alexithymia has also been considered a maladaptive and stable personality trait, Kleiger and Kinsman (1980) developed a scale assessing this phenomenon in the MMPI personality test. This self-report instrument consists of 22 items and allows clinicians to assess the subject’s affective denial characteristics, depletion of imagination, and tendency to confound feelings derived from emotional activation. However, the MMPI-alexithymia scale has shown poor internal validity, generating the need to put in place new standardized instruments to detect this characteristic (Bagby et al., 1991). Two other instruments were introduced: BIQ and SPSS. BIQ is a semi-structured interview developed by Sifneos (1973) and is the first valid attempt to assess alexithymia. The core items cover the following domains: verbalizing emotions, emotion expression, fantasy, and external thinking (Bermond et al., 2015). The second instrument (SPSS) derived from BIQ was the first self-report instrument to measure alexithymia (Apfel and Sifneos, 1979). This instrument has no specific factorial scales and offers only a global score, although a division into three scales can be traced in the literature: the ability to identify and describe feelings, the ability to fantasize, and the presence of apathy (Martin et al., 1984).

### 3.2. Presence of alexithymia in asthmatic populations

Among the selected studies, 16 (Kleiger and Jones, 1980; Dirks et al., 1981; Smyth et al., 2002; Plaza et al., 2006; Serrano et al., 2006; Chugg et al., 2009; Vázquez et al., 2010a; Baiardini et al., 2011; Barbosa et al., 2011; Moes-Wójtowicz et al., 2012; Amore et al., 2013; Innamorati et al., 2015; Khosravani et al., 2016, 2020; Vanegas et al., 2020; Dafauce et al., 2021) provided data on prevalence estimation of alexithymia in asthma patients, reporting different results based on the instruments used and the populations considered. Seven studies (Kleiger and Dirks, 1980; Brown et al., 1981; Feiguine et al., 1982; Nielsen et al., 1997; Feldman et al., 2002; Vazquez et al., 2010b; Liotta et al., 2021) reported the presence of alexithymia among asthmatic patients, without providing prevalence estimates or comparisons on mean alexithymia scores among patients with asthma or other controls. Three studies (Vliet et al., 2002; Martínez-Rivera et al., 2011; Ghorbani et al., 2017) compared the mean alexithymia scores of subjects with control groups with or without asthma.

The highest percentage was reported by Khosravani et al. (2016) in a sample of 300 Iranian asthma patients (62.8%), using the TAS-20. However, the authors stated that subjects with asthma presented lower levels of education than the control group, and the sample consisted of subjects with severe (108), moderate (139), and mild asthma (53). In a second study, Khosravani et al. (2020) revealed an alexithymia prevalence of 47% in a sample of 300 subjects with asthma; additionally, in this case, the number of patients with severe asthma (*n* = 134) was greater than that with mild discomfort (*n* = 32). According to these data, a high percentage of alexithymia (42,9%) was also reported by Dafauce et al. (2021), involving only patients with severe asthma. Liotta et al. (2021), who analyzed patients with severe allergic asthma (SAA), found that six out of 18 had a TAS-20 score of ≥ 61, indicating high-pathological levels of alexithymia. Therefore, the severity of asthma symptoms seemed to have a significant influence on the presence of alexithymia in patients with such chronic conditions (Liotta et al., 2021).

Amore et al. (2013) and Innamorati et al. (2015) found that 15 and 13.7% of patients with BA presented high TAS-20 scores (≥ 61), and these subjects also showed worse physical and mental health outcomes than asthmatic patients. Baiardini et al. (2011) reported that 19% of patients with asthma and comorbid rhinitis showed high levels of alexithymia, accompanied by a worsening of symptoms and lower quality of life.

With reference to NFA, Plaza et al. (2006) and Serrano et al. (2006), using the TAS-26, found that, respectively, 24 and 36% of NFA patients displayed high levels of alexithymia. On the contrary, Vázquez et al. (2010a), using TAS-20, reported 9% of 44 subjects with NFA presented higher levels of alexithymia than non-alexithymic subjects in the control group consisting of patients with asthma without any experience of near-fatal crisis. These differences could be due to the use of different versions of the TAS for the assessment of alexithymia in asthmatic patients.

Chugg et al. (2009) administered the TAS-20 to a sample of 25 patients with moderate-to-severe asthma and revealed that 12% were high alexithymic subjects and 52% had borderline scores. Using the same instrument, Vanegas et al. (2020) found that in large samples of Latin American subjects with asthma, 30.2% had high alexithymia scores. Barbosa et al. (2011) highlighted a higher prevalence of alexithymia in systemic lupus erythematosus patients (51%) than in a group of asthmatic subjects (34%), but differences in the mean scores were not significant. The authors concluded that difficulty in recognizing and decoding feelings may be a common factor in these chronic medical conditions. Smyth et al. (2002) found that 13% of participants had a high TAS-20 score (≥ 61). However, this study used a group consisting of subjects with asthma and rheumatoid arthritis, and the authors did not analyze the TAS-20 score differences between groups. Moes-Wójtowicz et al. (2012) used the TAS-26 with a sample of Polish asthmatic patients and reported high levels of alexithymia (21.6%).

Dirks et al. (1981) used MPPI-alexithymia scales with a sample of 549 patients receiving intensive treatment for asthma and found that 33.7% of the sample had high alexithymia and 47.8% presented borderline levels. This study considered alexithymia as a stable personality trait that can influence the life functioning areas of the subjects.

Finally, Kleiger and Jones (1980) studied a heterogeneous sample of subjects with chronic respiratory diseases, consisting mostly of patients with asthma. In this study, the authors revealed that 47% of subjects had alexithymia using a hetero-assessment tool (BIQ).

Regarding the remaining results, Ghorbani et al. (2017) found that asthmatic patients exhibited higher mean scores on the three alexithymia subscales of the TAS-20 that healthy controls. Martínez-Rivera et al. (2011) reported similar results, again using the TAS-20. Only Vliet et al. (2002) found no statistically significant differences in mean scores of the TAS-26 when comparing three groups of subjects (multiple chemical sensitivities, asthma, and healthy controls). However, there were fewer participants with asthma than in other studies, and data on the severity of disease were not provided.

### 3.3. Association between alexithymia and other factors intervening in asthma

Twenty-four studies (Kleiger and Dirks, 1980; Kleiger and Jones, 1980; Brown et al., 1981; Dirks et al., 1981; Feiguine et al., 1982; Nielsen et al., 1997; Feldman et al., 2002; Smyth et al., 2002; Plaza et al., 2006; Serrano et al., 2006; Chugg et al., 2009; Vázquez et al., 2010a; Vazquez et al., 2010b; Baiardini et al., 2011; Martínez-Rivera et al., 2011; Moes-Wójtowicz et al., 2012; Amore et al., 2013; Innamorati et al., 2015; Khosravani et al., 2016, 2020; Ghorbani et al., 2017; Vanegas et al., 2020; Dafauce et al., 2021; Liotta et al., 2021) reported associations among alexithymia and other factors implicated in asthma. The analysis of these scientific contributions highlights some relevant issues, including quality of life, control of symptoms, psychiatric morbidity, pulmonary functioning, and hospitalization.

All studies in which clinicians administrated instruments to measure quality of life in asthmatic subjects (Chugg et al., 2009; Vazquez et al., 2010b; Baiardini et al., 2011; Martínez-Rivera et al., 2011; Dafauce et al., 2021; Liotta et al., 2021) showed that alexithymia presented a significant influence on this variable, leading to a lower perceived quality of life, despite adjusting for confounders (i.e., sociodemographic characteristics and the presence of anxiety or depression). Martínez-Rivera et al. (2011), analyzing subscales of TAS-20, found that DIF was associated with a low control of asthma symptoms, anxiety, worse affective quality of life, and a high probability of developing dysfunctional breathing. Vazquez et al. (2010b) reported that DDF was associated with a worse physical dimension of quality of life, and this relationship was maintained even after controlling for depressive and anxious symptoms. It has been hypothesized that asthmatic patients who manifested this found it difficult to encode and express emotions, confused simple bodily sensations with asthma symptoms, and had a lower adherence and worsening general health (Vazquez et al., 2010b; Martínez-Rivera et al., 2011).

Asthma control represents the main treatment goal that will increase positive health outcomes. Eight studies (Serrano et al., 2006; Chugg et al., 2009; Baiardini et al., 2011; Martínez-Rivera et al., 2011; Moes-Wójtowicz et al., 2012; Amore et al., 2013; Dafauce et al., 2021; Liotta et al., 2021) investigated this factor in relation to alexithymia in subjects affected by asthma. Except for Moes-Wójtowicz et al. (2012), all the authors (Chugg et al., 2009; Baiardini et al., 2011; Martínez-Rivera et al., 2011; Amore et al., 2013; Vanegas., 2020; Dafauce et al., 2021; Liotta et al., 2021) found that alexithymia was associated with poor symptom control, and this finding was not directly related to the severity of asthma (Baiardini et al., 2011). Therefore, if on the one hand the presence of alexithymia was high in subjects with severe asthma (Khosravani et al., 2020; Vanegas et al., 2020; Dafauce et al., 2021), on the other hand, this variable acted as an independent factor on asthma management and symptom control (Baiardini et al., 2011).

Moes-Wójtowicz et al. (2012) did not report significant associations between alexithymia and asthma control. This result contrasts with data from other studies (Serrano et al., 2006; Chugg et al., 2009; Baiardini et al., 2011; Martínez-Rivera et al., 2011; Amore et al., 2013; Vanegas et al., 2020; Dafauce et al., 2021; Liotta et al., 2021); however, the sample size was limited, and the authors declared that subjects had difficulties in filling out the Asthma Control Test.

Psychopathological morbidity was analyzed in ten studies (Feldman et al., 2002; Plaza et al., 2006; Serrano et al., 2006; Vázquez et al., 2010a; Baiardini et al., 2011; Martínez-Rivera et al., 2011; Amore et al., 2013; Innamorati et al., 2015; Khosravani et al., 2020; Dafauce et al., 2021), with particular reference to depression and anxiety. Amore et al. (2013) and Innamorati et al. (2015) administered the State–Trait Anxiety Inventory (STAI) and Beck Depression Inventory (BDI), reporting different results. Amore et al. (2013) divided samples in terms of pulmonary functioning (high-low) and TAS-20 score (cutoff of > 61) and did not reveal differences in the levels of depression and anxiety, despite highlighting a significant correlation between alexithymia, depression, and anxiety. Innamorati et al. (2015) found high levels of alexithymia and low pulmonary functioning in a small group of patients exhibiting moderate-to-severe depression compared with other clusters of patients, but did not report differences between the groups in terms of anxiety levels. Baiardini et al. (2011), using an instrument that generally assesses emotional distress (Profile of Mood States), reported that alexithymic participants with asthma showed higher levels of depression (*p* < 0.001), confusion (*p* > 0.001), and inertia (*p* < 0.001) than non-alexithymic subjects. Dafauce et al. (2021), referring to patients with severe asthma, highlighted a high rate of anxiety, depression, alexithymia, and hyperventilation syndrome, with negative effects on global quality of life.

Feldman et al. (2002), when analyzing relationships between TAS-26 factors and self-report symptoms, found that DIF was associated with increased emotions and physical self-reported symptoms, and this relationship was mediated by trait anxiety, which was evaluated using the Taylor Manifest Anxiety Scale (TMAS). Khosravani et al. (2020), when administering Depression Anxiety Stress Scales (DASS) in patients with asthma of varying degrees, revealed that participants with high scores in DIF, DDF, and EOT showed higher DASS global scores. Furthermore, anxiety, depression, and emotional distress (DASS) represented a mediation factor between DIF and physical symptoms.

Two studies (Plaza et al., 2006; Serrano et al., 2006) that used the General Health Questionnaire (GHQ) to measure psychological health, indicated that psychiatric morbidity (anxiety and depression) was more frequent in subjects with life-threatening asthma with high alexithymia assessment (TAS-26) scores. Vázquez et al. (2010a) did not find differences in total TAS-20 scores between patients with NFA and control groups; however, they highlighted that DDF and anxiety-trait (STAI-T) influenced different health outcomes in both groups. Furthermore, in this study, the associations between alexithymia, asthma severity, and depression cognitive factors were not disclosed (Cognitive Depression Index, CDI).

Pulmonary functioning was evaluated through different methods in seven studies (Kleiger and Dirks, 1980; Feldman et al., 2002; Plaza et al., 2006; Serrano et al., 2006; Martínez-Rivera et al., 2011; Amore et al., 2013; Innamorati et al., 2015). Among these studies, three (Kleiger and Dirks, 1980; Plaza et al., 2006; Serrano et al., 2006) did not report significant differences in pulmonary parameters according to levels of alexithymia observed in patients with asthma. On the opposite side, four studies (Feldman et al., 2002; Martínez-Rivera et al., 2011; Amore et al., 2013; Innamorati et al., 2015) highlighted a relationship between the severity of alexithymia and impaired pulmonary function in asthma. Particularly, Feldman et al. (2002), administering the TAS-26, found that DDF was correlated with decreased pulmonary function. It was suggested that alexithymia might alter the ability of patients to communicate their experience of disease to clinicians, confounding sensations, emotions, and feelings related to their chronic condition, possibly underestimating the exacerbation of symptoms and compromising adherence and disease management (Chugg et al., 2009; Vanegas et al., 2020).

With regard to hospitalization, Dirks et al. (1981), evaluating a group of patients for 6 months, revealed a higher frequency of asthma-related hospitalizations in patients with severe alexithymia (37.4% vs. 28.4%) and also found a significant difference in mean days spent in hospice care (18,92 vs. 13,70) between patients with high levels of alexithymia and subjects with only asthma. Kleiger and Dirks (1980) analyzed the association between panic-fear personality, which is understood as the tendency to be overwhelmed and feeling helpless in the face of illness and alexithymia. Results indicated that independently of panic-fear personality, subjects who showed alexithymic personality trait (MMPI) had a higher frequency of previous hospitalizations. These results are in line with more recent studies (Plaza et al., 2006; Serrano et al., 2006; Vazquez et al., 2010b) that highlighted the increased need for emergency care in asthmatic patients with alexithymic characteristics.

Feiguine et al. (1982) analyzed alexithymia in relation to the age of asthmatic patients and found that the highest levels of alexithymia were present in middle (40–59) and late adulthood (> 65). Other studies confirmed these associations (Kleiger and Jones, 1980; Plaza et al., 2006; Serrano et al., 2006; Dafauce et al., 2021), showing that as age increases, levels of alexithymia tend to rise. Another interesting relationship emerged in relation to educational levels, as three studies (Serrano et al., 2006; Chugg et al., 2009; Vanegas et al., 2020) reported that alexithymia was higher in patients with a poor school education. Therefore, age and educational level may represent factors that increase levels of alexithymia with unfavorable outcomes in asthma.

Two studies conducted in Iran (Khosravani et al., 2016; Ghorbani et al., 2017) confirmed a close association between alexithymia, maladaptive emotional regulation strategies, and physical symptoms, including those not directly related to typical asthma manifestations. Furthermore, Ghorbani et al. (2017) highlighted that catastrophizing tendency was associated with all TAS-20 dimensions (DIF, DDF, and EOT) and was a mediator between alexithymia and physical symptoms. According to these results, Amore et al. (2013) found a correlation between alexithymia and avoidance coping strategies, with reference to denial and behavioral disengagement. These data suggested that alexithymia alters the ability of asthmatic patients to process emotional stimuli, producing negative effects on the psychological, physical, and social sphere of the individual.

Brown et al. (1981) found that alexithymic patients reported lower Asthma Symptoms Checklist scores and hypothesized that these individuals underestimate both physical and emotional components of asthma exacerbations. Kleiger and Jones (1980) reported that patients with alexithymia had high scores on the L scale and low scores on the psychasthenia scale of the MMPI. Additionally, in this case, the authors interpreted results while assuming that alexithymic individuals have greater difficulty in recognizing and processing their emotions.

Finally, two studies (Nielsen et al., 1997; Smyth et al., 2002) examined the association between alexithymia and particular aspects in patients with asthma. Nielsen et al. (1997) analyzed dream and nightmare recall and highlighted that the EOT factor, for men only, is associated with a lower memory of both. A study by Smyth et al. (2002) attempted to analyze the effect of different variables related to emotional processing on writing about traumatic events. In this case, there was no association between alexithymia and the ability to write about traumatic life events.

## 4. Discussion

Starting from the analysis of the scientific literature that focused on psychological factors influencing the course and management of asthma, it was hypothesized that alexithymia might negatively affect the health of patients. Therefore, the current Review attempted to detect the frequency of severe alexithymia in asthmatic populations and investigate the relationships among alexithymia and other factors involved in disease management. Evidence could provide new insights into the affective dynamics of patients with asthma, facilitating implementation of a multidisciplinary approach to the disease, improving the health of patients.

The term alexithymia has been translated from Greek to mean “wordlessness for emotions” (López-Muñoz and Pérez-Fernández, 2020). Since its introduction, this complex phenomenon has been investigated in chronic conditions (Martino et al., 2020a,b; Tang et al., 2022), psychosomatic disorders (Nakao and Takeuchi, 2018), and psychopathology (De Berardis et al., 2008; Sagar et al., 2021). Research has widely demonstrated that alexithymia may contribute to the impairment of biological, psychological, and social domains in individuals, increasing the risk of comorbidity and mortality among subjects affected by chronic diseases (Kojima et al., 2010; Kojima, 2012; Vadini et al., 2019).

Regarding chronic respiratory diseases, our results showed a high presence of alexithymia in patients with asthma, indicating a meaningful impairment of affective capacities and a tendency to confuse disease symptoms with emotional stimuli. The prevalence of alexithymia among the general population is estimated to be approximately 10% (Franz et al., 2008; Wang et al., 2021), while the results of the current Review indicate higher rates in asthma patient samples (from 9 to 62.7%).

However, among the considered studies, significant differences emerged based on disease severity, target population, geographic localization, and the psychodiagnostic instruments used to assess alexithymia, making a comparison difficult but stimulating useful considerations. It is an unmet need to provide additional empirical evidence on this topic, using a scientific multidisciplinary integrated approach that allows physicians and clinical psychologists to evaluate patient needs from an interdisciplinary perspective. Future research could promote a deeper understanding of the interrelationship between alexithymia and asthma, leading to the early identification of signs and symptoms and promoting better self-awareness and disease management and providing a higher perceived quality of life.

Moreover, concerning interrelationships among alexithymia and other factors involved in asthma management, the current Review highlighted that alexithymia is associated with a lower quality of life, low control of symptoms, a high number of hospitalizations, cognitive avoidance, and psychopathology. In addition, although the results are not always aligned, some studies have also found a significant association between alexithymia and impaired pulmonary function. As such, alexithymia represents a vulnerability factor in the management of chronic asthma and in the long-term health of the subject (Braido, 2013; Baiardini et al., 2015).

Our results are in line with the initial hypothesis and implement knowledge regarding the role of alexithymia in patients with asthma. Axelsson et al. (2009), in a study involving young adults with asthma, found that alexithymia was associated with a lower adherence to therapies, and Chung et al. (2012) suggested that this variable influenced the psychological health of subjects who had experienced an asthma crisis. These studies were not included in our review because the patient diagnoses were self-reported, so they did not meet the inclusion criteria, even if they add further empirical evidence to our findings. With reference to the relationship between alexithymia and medical conditions, Carta et al. (2022) reported that this variable is associated with an increased risk of death in a long-term course of myocardial infarction. Such an association may be due to a delay in requesting help, depending on the difficulty of the early identification of the typical symptoms of a heart attack. In accordance with these findings, our results showed that asthmatic patients with alexithymia have poor symptom control. Additionally, a high prevalence of alexithymia has been found in dermatological diseases (Holmes et al., 2022). In psoriasis (Panasiti et al., 2020) and atopic dermatitis (Talamonti et al., 2021), alexithymia has been associated with the presence of depression, difficult emotional regulation, and negative coping strategies (Founta et al., 2019; Belugina, 2021). In gastroenterology domains, a recent review on inflammatory bowel disease (Martino et al., 2020a) elucidated that the presence of alexithymia was correlated with symptoms of anxiety and depression. Kano et al. (2018), investigating the literature on functional gastrointestinal disorders (i.e., irritable bowel syndrome), found that alexithymia is associated not only with the visceral hypersensitivity that characterizes these conditions but also with an altered psychological processing of symptoms. However, the mechanisms of interaction between alexithymia and impaired gut-brain communication are still unclear. Nevertheless, alexithymia showed a significant role in other relevant clinical conditions, such as fibromyalgia and type 2 diabetes mellitus (T2DM). In particular, it has been highlighted that alexithymia increases the risk of psychological disorders in fibromyalgic patients, modifying the subjective experience of pain (Tesio et al., 2018; Marchi et al., 2019); additionally, alexithymia has been associated with poor glycemic control, difficulty in disease management, anxiety, and depression in T2DM patients (Martino et al., 2020b; Celik et al., 2022).

Researchers also investigated the relationship between alexithymia and chronic obstructive pulmonary disease (COPD), noting that this variable is associated with anxiety, depression, and impaired quality of life (Tselebis et al., 2010; Zhang et al., 2023), thus confirming the relevance of analyzing alexithymia in clinical settings.

Finally, the difficulties in recognizing and describing inner dynamics, feelings, and a poor imaginative life associated with operative thinking represent potential characteristics involved in the onset, maintenance, and unfavorable disease course (Lumley et al., 2008). Research highlights the existence of a subgroup of asthma patients presenting clinically significative levels of alexithymia. Such individuals show an inability to get in touch with emotions and feelings and poor interoceptive awareness (Kano and Fukudo, 2013). Therefore, this complex phenomenon could explain the difficulty in distinguishing states of emotional arousal from typical asthma symptoms (i.e., dyspnea, breathlessness, and asthma attack), with negative repercussions on disease self-management. From a clinical perspective, the present Review suggests the early identification of asthmatic patients presenting alexithymia is important. The psychodiagnostic path provides a solid basis that is useful for the evaluation of a patient’s psychological status, personological characteristics, and patient-tailored interventions and treatments.

Chronic disease, and in the specific case of asthma, due its characteristics, requires continuous adjustment for patients to balance daily life tasks with the demand of the illness. In this context, awareness of emotions and feelings and reflective ability occupy a fundamental role in ensuring wellbeing and equilibrium between these factors. Consistently, data from the current Review suggest that the negative impact of alexithymia on health may be effectively controlled through clinical intervention programs (Nunes da Silva, 2021; Zhang and Zhang, 2022). This path would favor the improvement of emotional skills, promotion of patient self-disclosure, and therapeutic alliance, driving toward a multi-integrated approach allowing early diagnosis by clinicians and collaborative treatment.

## 5. Strengths and limitations

To the best of our knowledge, this is the first systematic Review exploring the phenomenon of alexithymia in association with asthma. The current Review has several limitations. First, most of the studies taken into consideration used convenience samples; therefore, the severity and type of asthma were not equally distributed in the population. Based on the established criteria, not many studies could be included, limiting the possibility of generalizing our results to a broader context. In the included studies, the assessment of alexithymia was carried out with different instruments (TAS-20, TAS-26, MMPI, and BIQ), leading to heterogeneity in the results and limiting the possibility of making a quantitative synthesis. Based on the findings, the present Review is a first step toward a greater understanding of the alexithymia burden in asthma management and offers clinicians valuable information regarding the treatment of asthma patients.

## 6. Conclusion

This Review highlights a significant presence of alexithymia in asthma patients, in association with anxiety, depression, lower quality of life, difficulty in identifying asthma symptoms, maladaptive emotion regulation strategies, exacerbation of disease, and hospitalization. Therefore, clinicians and psychologists could apply a multidisciplinary approach to early detect the presence of alexithymia and asthma, improving the diagnostic and therapeutic work-up in clinical settings and providing a better perceived quality of life.

## Author contributions

GM and OS made significant contribution to the conception and design of the systematic review, to the acquisition, qualitative analysis, and synthesis of data by drafting both the first and revised versions of the manuscript. LR, AC, and CV contributed to the qualitative analysis and synthesis of data by drafting both the first and revised versions of the manuscript. FT, GP, GS, and PS gave significant contribution to draft part of the manuscript. PS, SG, and GM revised the manuscript for intellectual content and gave the final approval of the manuscript to be submitted. All authors contributed to the article and approved the submitted version.

## Conflict of interest

The authors declare that the research was conducted in the absence of any commercial or financial relationships that could be construed as a potential conflict of interest.

## Publisher’s note

All claims expressed in this article are solely those of the authors and do not necessarily represent those of their affiliated organizations, or those of the publisher, the editors and the reviewers. Any product that may be evaluated in this article, or claim that may be made by its manufacturer, is not guaranteed or endorsed by the publisher.
