# Supplementary material for: Alexithymia and asthma: a systematic review
Source: Front Psychol. 2023 Aug 7;14:1221648. doi: 10.3389/fpsyg.2023.1221648 (PMC10441120; doi:10.3389/fpsyg.2023.1221648)
Supplement: Supplementary file 1 [file Data_Sheet_1.docx]

**Supplementary** **material**

**Complete Search Strategy**

| **PubMed:** | ((Asthm* [all fields]) AND (alexithymia [all fields] OR "Toronto Alexithymia Scale" [all fields] OR "TAS-20"[all fields])) |
| --- | --- |

| **Scopus:** | ((asthm* OR asthma) AND (alexithymia OR “Toronto alexithymia scale” OR “tas-20”)) [Title, Abstract, Keywords] |
| --- | --- |
