# Supplementary material for: Alexithymia and asthma: a systematic review
Source: Front Psychol. 2023 Aug 7;14:1221648. doi: 10.3389/fpsyg.2023.1221648 (PMC10441120; doi:10.3389/fpsyg.2023.1221648)
Supplement: Supplementary file 2 [file Table_1.DOCX]

**Supplementary** **material**

**Table S1.** Studies were assessed using the NIH Quality Assessment Tool for Observational Cohort and Cross-sectional Studies.

| **Study** | **Was the research question or objective in this paper clearly stated?** | **Was the study population clearly specified and defined?** | **Was the participation rate of eligible persons at**  **least 50%?** | **Were all the subjects selected or recruited from the same or similar populations?** | **Was a sample size justification, power description, or variance and effect estimates provided?** | **For the analyses in this paper, were the exposure(s) of interest measured prior to the outcome(s) being measured?** | **Was the timeframe sufficient so that one could reasonably expect to see an association between exposure and outcome if it existed?** | **For exposures that can vary in amount or level, did the study examine different levels of the exposure?** | **Were the exposure measures (independent variables) clearly defined, valid, reliable, and implemented consistently across all study participants?** | **Was the exposure(s) assessed more than once over time?** | **Were the outcome measures (dependent variables) clearly defined, valid, reliable, and implemented? consistently across all study participants?** | **Were the outcome assessors blinded to the exposure status of participants?** | **Was loss to follow-up after baseline 20% or less?** | **Were key potential confounding variables measured and adjusted statistically for their impact on the relationship? between exposure(s) and outcome(s)?** | **Summary Quality** |
| --- | --- | --- | --- | --- | --- | --- | --- | --- | --- | --- | --- | --- | --- | --- | --- |
| Amore et al., 2013 | ✓ | ✓ | ✓ | ✓ | 🗶 | ✓ | 🗶 | ✓ | ✓ | NA | ✓ | NR | 🗶 | ✓ | i |
| Baiardini et al., 2011 | ✓ | ✓ | ✓ | ✓ | 🗶 | ✓ | ✓ | ✓ | ✓ | NR | ✓ | NR | ✓ | ✓ | ii |
| Barbosa et al., 2011 | ✓ | ✓ | ✓ | ✓ | 🗶 | ✓ | 🗶 | 🗶 | ✓ | 🗶 | ✓ | NR | NA | 🗶 | i |
| Brown et al.,  1981 | ✓ | ✓ | ✓ | ✓ | 🗶 | 🗶 | 🗶 | 🗶 | ✓ | 🗶 | ✓ | NR | NA | NR | i |
| Chugg et al.,  2009 | ✓ | ✓ | 🗶 | ✓ | 🗶 | ✓ | ✓ | ✓ | ✓ | 🗶 | ✓ | NR | NA | ✓ | i |
| Dafauce et al., 2021 | ✓ | ✓ | ✓ | ✓ | ✓ | ✓ | 🗶 | ✓ | ✓ | 🗶 | ✓ | NR | NA | ✓ | ii |
| Dirks et al., 1981 | ✓ | ✓ | ✓ | ✓ | 🗶 | ✓ | ✓ | ✓ | ✓ | ✓ | ✓ | NR | ✓ | 🗶 | ii |
| Feiguine et al., 1982 | ✓ | ✓ | ✓ | ✓ | 🗶 | ✓ | 🗶 | ✓ | ✓ | 🗶 | ✓ | NR | NA | ✓ | i |
| Feldman et al., 2002 | ✓ | ✓ | NR | ✓ | 🗶 | ✓ | 🗶 | ✓ | ✓ | NA | ✓ | 🗶 | NA | ✓ | i |
| Ghorbani et al., 2017 | ✓ | ✓ | ✓ | ✓ | ✓ | ✓ | 🗶 | ✓ | ✓ | 🗶 | ✓ | 🗶 | NA | ✓ | ii |
| Innamorati et al., 2015 | ✓ | ✓ | ✓ | ✓ | 🗶 | ✓ | 🗶 | ✓ | ✓ | NA | ✓ | NR | NA | ✓ | i |
| Khosravani et al., 2016 | ✓ | ✓ | ✓ | ✓ | ✓ | ✓ | 🗶 | ✓ | ✓ | 🗶 | ✓ | 🗶 | NA | ✓ | ii |
| Khosravani et al., 2020 | ✓ | ✓ | ✓ | ✓ | ✓ | ✓ | 🗶 | ✓ | ✓ | 🗶 | ✓ | NR | NA | ✓ | ii |
| Kleiger & Dirks 1980 | ✓ | ✓ | ✓ | ✓ | 🗶 | ✓ | ✓ | ✓ | ✓ | ✓ | ✓ | NR | ✓ | 🗶 | ii |
| Kleiger & Jones 1980 | ✓ | ✓ | ✓ | ✓ | 🗶 | ✓ | 🗶 | ✓ | ✓ | 🗶 | ✓ | 🗶 | NA | 🗶 | i |
| Martínez-Rivera et al. 2011 | ✓ | ✓ | NR | ✓ | 🗶 | ✓ | 🗶 | ✓ | ✓ | NA | ✓ | NR | NA | ✓ | i |
| Moes-Wójtowicz et al. 2012 | ✓ | ✓ | NR | ✓ | 🗶 | ✓ | 🗶 | ✓ | ✓ | 🗶 | ✓ | 🗶 | NA | 🗶 | i |
| Nielsen et al., 1997 | ✓ | ✓ | NR | ✓ | 🗶 | ✓ | 🗶 | 🗶 | ✓ | 🗶 | 🗶 | NR | NA | ✓ | i |
| Plaza et al., 2006 | ✓ | ✓ | NR | ✓ | 🗶 | ✓ | 🗶 | ✓ | ✓ | 🗶 | ✓ | NR | NA | 🗶 | i |
| Serrano et al., 2006 | ✓ | ✓ | 🗶 | ✓ | 🗶 | ✓ | 🗶 | 🗶 | ✓ | 🗶 | ✓ | NR | NA | ✓ | i |
| Vanegas et al., 2020 | ✓ | ✓ | ✓ | ✓ | ✓ | ✓ | 🗶 | ✓ | ✓ | NA | ✓ | NR | NA | ✓ | ii |
| Vasquez et al., 2010a | ✓ | ✓ | NR | ✓ | 🗶 | ✓ | 🗶 | ✓ | ✓ | 🗶 | ✓ | NR | NA | ✓ | i |
| Vasquez et al., 2010b | ✓ | ✓ | NR | ✓ | 🗶 | ✓ | ✓ | ✓ | ✓ | 🗶 | ✓ | NR | 🗶 | ✓ | i |
| Vliet et al., 2002 | ✓ | ✓ | NR | ✓ | 🗶 | ✓ | 🗶 | ✓ | ✓ | 🗶 | ✓ | NR | NA | ✓ | i |

Quality was rated as 0 for **poor** (0–4 out of 14 questions), **i** for **fair** (5–10 out of 14 questions), or **ii** for **good** (11–14 out of 14 questions); **NA**: not applicable, **NR**: not reported.
