# Supplementary material for: Alexithymia and asthma: a systematic review
Source: Front Psychol. 2023 Aug 7;14:1221648. doi: 10.3389/fpsyg.2023.1221648 (PMC10441120; doi:10.3389/fpsyg.2023.1221648)
Supplement: Supplementary file 3 [file Table_2.DOCX]

**Supplementary** **material**

**Table S2.** Studies were assessed using the NIH Quality Assessment Tool for Before-After (Pre-Post) Studies with No Control Group.

| **Study** | **Was the study question or objective clearly stated?** | **Were eligibility/selection criteria for the study population prespecified and clearly described?** | **Were the participants in the study representative of those who would be eligible for the test/service/intervention in the general or clinical population of interest?** | **Were all eligible participants that met the prespecified entry criteria enrolled?** | **Was the sample size sufficiently large to provide confidence in the findings?** | **Was the test/service/intervention clearly described and delivered consistently across the study population?** | **Were the outcome measures prespecified, clearly defined, valid, reliable, and assessed consistently across all study participants?** | **Were the people assessing the outcomes blinded to the participants' exposures/interventions?** | **Was the loss to follow-up after baseline 20% or less? Were those lost to follow-up accounted for in the analysis** | **Did the statistical methods examine changes in outcome measures from before to after the intervention?** | **Were outcome measures of interest taken multiple times before the intervention and multiple times after the intervention?** | **If the intervention was conducted at a group level did the statistical analysis take into account the use of individual-level data to determine effects at the group level?** | **Summary Quality** |
| --- | --- | --- | --- | --- | --- | --- | --- | --- | --- | --- | --- | --- | --- |
| Liotta et al., 2021 | ✓ | ✓ | ✓ | ✓ | 🗶 | ✓ | ✓ | NR | ✓ | ✓ | ✓ | NA | ii |

Quality was rated as 0 for **poor** (0–4 out of 12 questions), **i** for **fair** (5–9 out of 14 questions), or **ii** for **good** (10–12 out of 14 questions); **NA**: not applicable, **NR**: not reported.
