# Supplementary material for: Alexithymia and asthma: a systematic review
Source: Front Psychol. 2023 Aug 7;14:1221648. doi: 10.3389/fpsyg.2023.1221648 (PMC10441120; doi:10.3389/fpsyg.2023.1221648)
Supplement: Supplementary file 4 [file Table_3.DOCX]

**Supplementary** **material**

**Table S3.** Studies were assessed using the NIH Quality Assessment of Controlled Intervention Studies.

| **Study** | **Was the study described as randomized, a randomized trial, a randomized clinical trial, or an RCT?** | **Was the method of randomization adequate (i.e., use of randomly generated assignment)?** | | **Was the treatment allocation concealed (so that assignments could not be predicted)?** | **Were study participants and providers blinded to treatment group assignment?** | **Were the people assessing the outcomes blinded to the participants' group assignments?** | **Were the groups similar at baseline on important characteristics that could affect outcomes (e.g., demographics, risk factors, co-morbid conditions)?** | **Was the overall drop-out rate from the study at endpoint 20% or lower of the number allocated to treatment?** | **Was the differential drop-out rate (between treatment groups) at endpoint 15 percentage points or lower?** | **Was there high adherence to the intervention protocols for each treatment group?** | **Were other interventions avoided or similar in the groups (e.g., similar background treatments)?** | **Were outcomes assessed using valid and reliable measures, implemented consistently across all study participants?** | **Did the authors report that the sample size was sufficiently large to be able to detect a difference in the main outcome between groups with at least 80% power?** | **Were outcomes reported or subgroups analyzed prespecified (i.e., identified before analyses were conducted)?** | **Were all randomized participants analyzed in the group to which they were originally assigned, i.e., did they use an intention-to-treat analysis?** | **Summary Quality** |
| --- | --- | --- | --- | --- | --- | --- | --- | --- | --- | --- | --- | --- | --- | --- | --- | --- |
| Smyth et al., 2002 | ✓ | ✓ | ✓ | | ✓ | 🗶 | ✓ | ✓ | ✓ | ✓ | ✓ | ✓ | 🗶 | ✓ | ✓ | ii |

Quality was rated as 0 for **poor** (0–4 out of 14 questions), **i** for **fair** (5–10 out of 14 questions), or **ii** for **good** (11–14 out of 14 questions); **NA**: not applicable, **NR**: not reported.

NIH quality assessment tool was available at: <https://www.nhlbi.nih.gov/health-topics/studyquality-assessment-tools>
